# Whole steamed corn enhances growth performance and alters rumen microbiota in fattening lambs

**DOI:** 10.5713/ab.250524

**Published:** 2025-11-10

**Authors:** Yuhua He, Xuezhao Sun

**Affiliations:** 1 College of Animal Science and Technology, Jilin Agricultural Science and Technology College, Jilin, China; 2 Jilin Inter-regional Cooperation Centre for the Scientific and Technological Innovation of Ruminant Precision Nutrition and Smart and Ecological Farming, Jilin, China; 3 Bioeconomy Science Institute, Grasslands Research Centre, Palmerston North, New Zealand

**Keywords:** Corn Processing, Digestibility, Growth Performance, Lamb Fattening, Rumen Microbiota, Steam Cooking

## Abstract

**Objective:**

To evaluate the effects of four processing methods: intact corn (IT), ground corn (GD; ground to 2 mm), steam-flaked corn (SF; steamed at 85°C–100°C for 90 min and flaked to 2.5 mm thickness), and whole steamed corn (WS; steamed at 85°C–100°C for 90 min without flaking), on growth performance, digestibility, blood biochemistry, rumen microbiota, carcass traits, and meat quality in fattening lambs.

**Methods:**

Eighty-four male crossbred lambs (Small tailed Han×Northeastern Fine wool; 4.5 months; 34.2±3.5 kg) were blocked by stratifying body weight and then randomly assigned to four treatments (n = 21), each with 50% (as-fed) corn in the diet. In GD and WS treatments, corn was pelleted with other ingredients; in IT and SF treatments, corn was fed separately alongside non-corn pellets at a 1:1 (as-fed) ratio. After a 14 d adaptation period, lambs were fed for 60 d. Body weights were recorded on d 0, 30, and 60; blood samples were collected on d 31 and 60; and rumen fluid was sampled on d 35. Apparent total tract digestibility was determined by total fecal collection in a subset of lambs, and six lambs per treatment were slaughtered for carcass evaluation.

**Results:**

Lambs fed the WS diet showed the greatest average daily gain (ADG) at 319 g/d, improving 22%, 24%, and 7% over GD (261 g/d), SF (257 g/d), and IT (298 g/d) diets, respectively (p = 0.013). Dry matter intake did not differ significantly among treatments (p = 0.307), though WS, GD, and IT numerically exceeded SF by 6.7%–10.8%. Apparent total tract dry matter digestibility was greatest in SF (74.1%) and WS (72.1%), exceeding GD (69.3%) and IT (66.3%; p = 0.001) for the overall diet. Corn processing also altered rumen microbiota: WS tended to increase *Erysipelotrichaceae*_UCG 002 abundance sixfold over IT, SF enriched *Actinobacteria*, and IT uniquely harbored minor phyla such as *Acidobacteriota*. Hot carcass weight tended to be higher in WS (18.4% over GD; p = 0.078), while heart and kidney indices were greater in IT and SF (p<0.05). Meat quality parameters were not significantly affected by corn processing.

**Conclusion:**

Whole steamed corn enhanced growth performance, likely associated with improved starch utilization and modulation of the rumen microbiota, without compromising meat quality. Compared with grinding or flaking, steaming corn kernels provided a practical and energy-efficient approach, yielding 22%–24% higher ADG. Further research should refine steaming conditions and evaluate the economic viability of this method.

## INTRODUCTION

Corn is a major energy source in lamb fattening diets, primarily because of its high starch content, which can comprise over half the diet [1,2]. However, rapid ruminal starch degradation can generate excessive short-chain fatty acids, lowering rumen pH and increasing the risk of acidosis [3]. This condition compromises lamb health [4], and leads to energy losses as starch is fermented by rumen microbes rather than absorbed directly [5].

In contrast, rumen bypass starch escapes ruminal degradation and is digested in the small intestine, where it is converted to glucose and absorbed directly [6]. This pathway is more energy-efficient than microbial fermentation [3], supporting better feed efficiency, average daily gain (ADG), and nutrient utilization, while reducing the risks of acidosis, diarrhea, and colonic inflammation [7,8].

The outer layers of corn kernels, including the cuticular wax and pericarp, naturally act as physical barriers limiting microbial access to starch, thereby increasing the proportion of bypass starch in unprocessed kernels. Processing methods such as grinding, flaking, and steaming disrupt these barriers, making starch more available to rumen microbes [9]. In addition, corn starch is embedded in a prolamin (zein) protein matrix [10], which must be altered to improve digestibility [7]. Depending on processing conditions, 18%–42% of dietary starch may bypass ruminal fermentation and be digested post-ruminally [11,12]. Ground and flaked corn accelerate ruminal starch degradation, whereas whole corn slows fermentation and increases bypass starch [13].

Steam flaking (steam-flaked corn [SF]; steamed at 85°C–100°C for 90 min and flaked to 2.5 mm thickness), widely used in beef cattle diets, gelatinizes starch and improves digestibility in both the rumen and small intestine [14]. However, excessive ruminal starch digestion reduces bypass starch, increasing fermentation losses. While ruminal digestion contributes microbial protein, starch digestion in the small intestine is more energy-efficient as it avoids these losses [3,6]. Therefore, an optimal processing method should increase post-ruminal starch availability without excessively accelerating ruminal degradation. Steam flaking improves starch digestibility but decreases bypass starch [14]. In this context, steaming intact kernels without flaking may partially gelatinize starch, enhancing its utilization in the small intestine while preserving kernel integrity to sustain bypass starch. Nonetheless, steam flaking requires 30%–40% more electrical and thermal energy per ton compared with dry rolling or grinding [15].

In sheep, steam-flaked corn has not consistently improved production performance relative to whole corn [16]. Furthermore, the optimal flake density for feedlot cattle (310–360 g/L) may not be suitable for sheep, and over-processing to achieve these densities can reduce feed intake and mill throughput [15]. Thus, while steam flaking is well established for beef cattle, its advantages in lamb fattening remain uncertain. In contrast, feeding intact whole grains (intact corn [IT]) promotes rumination and saliva secretion, which buffer rumen pH and improve feed efficiency [17]. Recent studies have reported that steam-flaking and whole-grain cooking increase starch degradability, nutrient utilization, and reproductive outcomes in ewes [16]. However, the effects of steaming whole corn without flaking have not been clearly defined, particularly in lamb fattening systems.

Given the increasing reliance on corn in intensive lamb production, optimizing starch utilization is critical. We propose an alternative method: whole steamed corn (WS), in which whole kernels are steam-cooked without flaking. Steaming disrupts the protein matrix and gelatinizes starch granules, enhancing enzymatic accessibility in the small intestine, while the absence of flaking preserves particle size to increase bypass starch, enhance energy efficiency, and reduce acidosis risk. However, the impacts of this method on lamb performance, nutrient digestion, and rumen microbiota remain unclear.

Therefore, the objective of this study was to compare four corn processing methods: IT, SF, WS, and ground corn (GD), on lamb growth performance, nutrient digestibility, blood biochemical indices, microbial composition, carcass traits, and meat quality. We hypothesize that WS, by combining starch gelatinization with kernel integrity, would balance digestibility and bypass starch, improving post-ruminal starch utilization and growth performance while reducing processing energy requirements and the risk of acidosis.

## MATERIALS AND METHODS

### Experimental design, animals, and diet

Four dietary treatments differing in corn processing methods were evaluated: WS, GD, IT, and SF. Corn accounted for 50% of the total mixed ration (as-fed basis) in all treatments.

A total of 84 crossbred male lambs (Small-tailed Han sheep×Northeastern Fine-wool sheep) with an average age of 4.5 months and an initial body weight (BW) of 34.2±3.5 kg were used. After a 14-d adaptation period, lambs were stratified by BW into three blocks of 28 animals each. Within each block, lambs were randomly assigned to one of the four treatments, with seven lambs per treatment per block housed in a single pen. The feeding trial lasted 60 d.

Lambs were weighed on d 0, 30, and 60 before morning feeding. On d 31 and 60, blood samples were collected before morning feeding from four randomly selected lambs per pen (n = 12 per treatment). Rumen fluid was collected on d 35 before morning feeding from five randomly selected lambs per pen (n = 15 per treatment) to characterize rumen microbiota after adaptation but before final growth measurements. At the end of the 60-d feeding period, two lambs per pen (n = 6 per treatment) were randomly selected for slaughter, and another two lambs per pen (n = 6 per treatment) were randomly selected for a digestibility trial consisting of a 6-d adaptation and 7-d formal measurement period.

Diets were formulated according to the Chinese Feeding Standard for Fattening Lambs (NY/T 816–2004) [18]. The ingredient composition and nutritional levels are presented in Table 1.

In the GD treatment, corn was ground using a hammer mill fitted with a 2-mm screen. Other ingredients (corn germ meal, sunflower meal, rice husk, sunflower seed hulls, and cottonseed meal) were ground using a 4/6-mm screen (half 4-mm, half 6-mm holes) and then mixed with premix, bentonite, limestone, dicalcium phosphate, and sodium chloride. Corn and other ingredients were combined at a 1:1 (as-fed) ratio and pelleted under the following conditions: conditioning at 85°C for 45 s, pelleting at 90°C, air cooling, and a ring die compression ratio of 1:7.

In the IT treatment, IT was used without processing. Non-corn ingredients were pelleted using the same machine under the same conditions as the GD treatment. These non-corn pellets were mixed with the whole corn at a 1:1 (as-fed) ratio at feeding.

In the SF treatment, corn was processed using a steam flaker (30T/D; Shandong GuanFeng Machinery), steamed at 85°C–100°C for 90 min, flaked to 2.5 mm thickness, and cooled. SF was mixed 1:1 (as-fed) with the non-corn pellets at feeding.

In the WS treatment, whole corn was steamed under the same conditions as the SF treatment, but the roller gap was widened to 15 mm to maintain kernel integrity. The WS was mixed 1:1 (as-fed) with the other ingredients and pelleted under the same conditions as the GD treatment. After pelleting, some corn kernels in the pellets were partially broken rather than intact.

All feed pellets (5 mm in diameter, 8–10 mm in length) were produced in a single batch at the Chifeng Branch of Jiangsu AgriPortal Feed using a pelleting machine (YPM508E; Jiangsu Yongli Machinery). Pellets were stored in moisture-proof bags away from light.

### Animal management and sampling

The experiment was conducted at the Experimental Station of the College of Animal Science and Technology, Jilin Agricultural Science and Technology College. Lambs were housed in a semi-open barn with an earthen floor, brick side walls, and a roof of plastic sheeting covered with shade netting. All lambs were dewormed by subcutaneous injection of ivermectin in the neck (0.7 mL; Jilin Yige-Feng Animal Pharmaceutical) [20].

Feed was offered twice daily at 08:30 and 16:00 h in troughs in each pen. Refusals were maintained at 5%–10% of the amount offered to ensure *ad libitum* intake. Water was available at all times. Refusals were collected daily before morning feeding, weighed, and dried at 65°C for 48 h to determine dry matter intake.

A mineral lick block was provided in each trough, containing iron (800–1,500 mg/kg), manganese (600–1,000 mg/kg), zinc (1,000–1,800 mg/kg), sodium chloride (850–920 g/kg), magnesium (6–9 g/kg), phosphorus (20–60 mg/kg), calcium (50–120 mg/kg), and copper (8–30 mg/kg).

Residual feed was removed at 20:00 h the evening before BW weighing, and water was withheld for 2 h before weighing. Body weight was measured before morning feeding using an electronic scale (accuracy ±0.05 kg; Dahe Weighing Instrument Factory). ADG was calculated from the slope of BW over time.

Blood was collected from the jugular vein after 12-h fasting using 5 mL vacutainer tubes (Hebei Xinle Medical Instrument Technology). Samples were centrifuged at 2,770×g for 10 min (TDL-40B; Shanghai Anting), and serum was transferred to 1.5 mL microcentrifuge tubes and stored at −80°C for biochemical analysis.

Rumen fluid was collected using a stomach tube, discarding the first 30–50 mL to minimize saliva contamination [21]. The pH was measured immediately using a pH meter (PHSJ-3F; Shanghai Instrument and Electric Instrument). Samples were placed in 50 mL centrifuge tubes, kept on ice, and transported to the laboratory within 30 min. Aliquots were transferred into three 10 mL cryotubes and stored at −80°C for microbiota analysis.

### Digestibility trial

Apparent nutrient digestibility was determined by the total fecal collection [22]. Lambs were housed individually in metabolic cages and fitted with fecal collection bags the day before sampling. Feces were collected daily for seven consecutive days following a 6-day adaptation period. Feed refusals were collected daily, stored in resealable plastic bags, and kept at −20°C. After the collection period, feces and refusals were pooled by animal and oven-dried at 65°C to constant weight using an electric drying oven (DHG-9203A; Shanghai Qixin Scientific Instrument).

### Slaughter trial

At the end of the feeding period, selected lambs were fasted for 24 h (water available) before slaughter. Pre-slaughter BW, hot carcass weight (HCW), and the weights of the head and hooves were recorded to calculate dressing percentage.

Carcass traits, including GR tissue depth (an indicator of fat depth), eye muscle area (EMA), and backfat thickness, were assessed. GR tissue depth was measured between the 12th and 13th ribs, 11 cm lateral to the dorsal midline. EMA was determined by tracing the cross-section of the *longissimus dorsi* muscle between the 12th and 13th ribs and calculated using: EMA (cm^2^) = muscle height (cm)×muscle width (cm)× 0.7. Backfat thickness was measured on the left half of the carcass between the 12th and 13th ribs using a vernier caliper (Model 150–200–300; Hangzhou Hailin Tools) following the method of Liu et al [23].

Hearts, livers, spleens, lungs, and kidneys were excised, trimmed of connective and adipose tissues, and weighed using an electronic balance (EX35001ZH; Ohaus International Trading). Organ indices were calculated as organ weight/BW (g/kg). The pH of rumen, duodenal, jejunal, ileal, and cecal contents was measured using a pH meter (PHSJ-3F; Shanghai Instrument and Electric Instrument) immediately after collection post-mortem. Approximately 1,000 g of *longissimus dorsi* muscle was collected from each carcass for meat quality analysis.

### Laboratory analyses

Dry matter (DM) in feed and feces, as well as crude protein (CP), neutral detergent fiber (NDF), acid detergent fiber (ADF), ash, calcium, and phosphorus (P) in feed, were analyzed as described by Q Huo et al [24].

Serum biochemical parameters, including alanine aminotransferase (ALT), aspartate aminotransferase (AST), alkaline phosphatase (ALP), total protein (TP), albumin (ALB), creatinine (CREA-J), urea (UREA), total cholesterol (TC), triglycerides (TG), high-density lipoprotein cholesterol (HDL-C), low-density lipoprotein cholesterol (LDL-C), glucose (Glu-O), α-amylase (α-AMY), lipase (LIP), and non-esterified fatty acids (NEFA), were measured using an automated biochemical analyzer (BS-350; Mindray Bio-Medical Electronics). NEFA assay kits were obtained from Nanjing Jiancheng Bioengineering Institute; other reagents were from Mindray Bio-Medical Electronics.

Marbling was scored at the *longissimus dorsi* cross-section between the 12th and 13th ribs using a marbling reference card (US NPPC standard; Beijing Tianxiang Feiyu Instrument Equipment). Cooking loss was determined by weighing approximately 100 g of *longissimus dorsi*, steaming at 70°C for 50 min (RT2169; Midea Group), cooling, and reweighing. Results were expressed as the percentage of weight loss after cooking [25]. Shear force was measured on strips (3.0 cm×1.0 cm×1.0 cm) cut parallel to muscle fibers using a tenderness meter (C-LM3B; Northeast Agricultural University Engineering College) [26].

For rumen and reticulum mucosal morphology, 3–5 tissue samples per lamb were fixed in 4% formaldehyde. Five 1 cm^2^ sections per sample were prepared to measure ruminal mucosal thickness, papilla density, papilla length and width, and reticulum mucosal thickness using calipers (150–200–300; Hangzhou Hailing Tools) and forceps.

Rumen microbiota analysis followed B Li et al [27]. Genomic DNA was extracted from rumen content using the CTAB method, verified by gel electrophoresis, and diluted to 1 ng/μL. The V4–V5 region of the 16S rRNA gene was amplified with barcoded primers, purified, and sequenced on an Illumina NovaSeq6000. Raw reads were merged, quality-filtered, and chimera-checked, then clustered into operational taxonomic units (OTUs) at 97% identity. Representative sequences were assigned taxonomy using the SILVA database (v138) (https://www.arb-silva.de/) and aligned for phylogenetic analysis. Data were rarefied to equal sequencing depth, and alpha (Observed OTUs, Chao1, Shannon) and beta diversity (UniFrac distances, ordination) metrics were calculated, and differential taxa were identified using LEfSe with false discovery rate correction.

### Statistical analysis

Except blood and microbiota data, all variables were analyzed using one-way analysis of variance (ANOVA) using treatment as a fixed effect and block as a random effect. Blood biochemical parameters were analyzed by two-way mixed-effects ANOVA with treatment and sampling time as fixed effects and block as a random effect. Statistical analyses were performed using GenStat (22nd edition; VSN International) [28]. When significant effects were detected, mean comparisons were conducted using pairwise t-tests with Bonferroni correction.

Microbial relative abundances at phylum and genus levels were analyzed using non-parametric methods due to non-normal distributions, as confirmed by Shapiro-Wilk tests. Differences among treatments were assessed using the Kruskal-Wallis test followed by Dunn’s post hoc test with Bonferroni correction. Microbiota analyses were performed in Python (v3.12) using the scipy and scikit-posthocs packages. Results are expressed as mean±standard error, and differences were considered significant at p<0.05.

## RESULTS

### Animal performance and dry matter digestibility

Initial BW was similar among treatments, with a variation of less than 2.3% (Table 2; p = 0.772). However, ADG was significantly affected by corn processing method (p = 0.013). Lambs fed the WS diet achieved the greatest ADG, significantly exceeding those fed GD and SF diets by 22% (58 g/d) and 24% (62 g/d), respectively. The ADG of lambs fed the IT diet was intermediate and did not differ significantly from other treatments.

Dry matter intake (DMI) was not affected by treatment (p = 0.307). Nevertheless, DMI was numerically higher in WS, GD, and IT treatments than in SF treatment by 10.8%, 6.7%, and 8.8%, respectively. Feed conversion efficiency, expressed as the feed-to-gain ratio, was significantly improved in the WS treatment (4.85) compared with the GD treatment (5.71; p<0.05), while IT and SF treatments were intermediate. Overall, WS improved feed efficiency by approximately 5%–11% relative to the other treatments.

DM digestibility was also influenced by processing method (p = 0.001). The highest digestibility was observed in SF, followed by WS, GD, and IT. The digestibility of the WS diet was 2.8 and 5.8 percentage units higher than in GD and IT diets, respectively.

### Blood biochemical parameters

Blood biochemical profiles (Table 3) indicated that TP was affected by processing method (p = 0.022), with the GD treatment showing the highest concentrations compared with IT and SF treatments, while the WS treatment was intermediate. Sampling time (d 31 vs. d 60) significantly influenced ALB (p<0.001), blood urea nitrogen (BUN; p<0.001), glucose (p = 0.022), cholesterol (p<0.001), and AST (p<0.001), with no significant treatment×time interactions.

Corn processing also influenced serum lipid metabolism. Cholesterol concentration differed among treatments (p = 0.004), being lowest in SF (1.47 mmol/L) compared with 1.71–1.86 mmol/L in the other treatments. Similarly, LDL concentrations were lower in SF (0.536 mmol/L) than in the other treatments (0.614–0.712 mmol/L; p = 0.029). Processing had no significant effects on α-AMY, LIP, ALT, or ALP.

### Rumen microbial communities

At the phylum level, Bacteroidota predominated across all treatments, comprising ~48% of total sequences, with no significant treatment effects (Table 4). Firmicutes was the second most abundant phylum and differed significantly among treatments (p = 0.028), being lowest in IT (26.3%) and higher in WS, GD, and SF (39.9%–42.9%), representing a 52%–63% increase relative to IT, though these three treatments did not differ significantly. The ratio of Firmicutes to Bacteroidota was also higher in WS, GD, and SF than in IT (p = 0.023) and WS was numerically lower than GD and SF. Unidentified_Bacteria was most abundant in IT and least abundant in GD. Actinobacteria were more abundant in SF than in the other treatments (p = 0.018). Minor phyla, including Acidobacteriota, Chloroflexi, WPS-2, and RCP2-54, were detected primarily in the rumen of lambs fed the IT diet, each contributing 0.2%–1.2% of total abundance (p<0.001).

At the genus level, distinct patterns were observed. In the IT treatment, *Succinivibrionaceae_UCG-001* (3.8%; p = 0.005) and *Rikenellaceae_RC9_gut_group* (2.8%; p = 0.010) were more abundant than in WS and GD treatments. The IT lambs also uniquely hosted several low-abundance genera, including *Acidothermus*, *Puia*, *Acidibacter*, *Candidatus_Solibacter*, and *Bryobacter* (each 0.2%–0.3%; p<0.001). In the SF treatment, *Pseudoscardovia* (1.9%; p = 0.016) was most abundant, along with higher relative abundances of *Megasphaera* (0.8%; p = 0.003) and *Oribacterium* (0.3%; p = 0.020). The WS treatment showed a markedly higher abundance of *Erysipelotrichaceae*_UCG-002, over sixfold that of IT (p = 0.066), similar to GD and SF. The GD treatment exhibited the lowest abundance of *Olsenella*.

### Slaughter performance, meat quality, and rumen morphology

Slaughter performance parameters, including final BW, HCW, dressing percentage, subcutaneous fat depth, EMA, and backfat thickness, were not significantly affected by corn processing method (Table 5; p>0.05). However, HCW tended to differ among treatments (p = 0.078), with WS being 18% greater than GD.

Organ weights varied significantly among treatments. Heart and kidney weights were higher in IT and WS than in GD and SF. Correspondingly, heart and kidney indices were greater in IT (0.45% and 0.31%; p<0.05). Gastrointestinal pH values did not differ significantly across most segments of the digestive tract, including the rumen (pH 5.88–6.38), although duodenal pH tended to be higher in IT than in SF (p = 0.079).

Meat quality parameters, including tenderness, marbling score, and cooked meat yield, were unaffected by corn processing (p>0.05). Similarly, ruminal histomorphological characteristics (wall thickness, papillae number, length, width, and reticulum wall thickness) did not differ among treatments (Table 6; all p>0.35).

## DISCUSSION

The four corn processing strategies evaluated in this study were selected to represent the range of physical and thermal processing methods available to commercial sheep producers. Whole steamed corn was included because moist-heat treatments can gelatinize starch while maintaining kernel integrity, thereby improving feed conversion under certain conditions in ruminants [29]. Fine grinding represented the conventional mill-to-pellet practice, which increases surface area and ruminal fermentation rate but may predispose animals to subacute ruminal acidosis [30]. Intact corn was included as a “minimal-processing” negative control to capture the upper limit of particle-size effects without thermal input [31]. Feeding whole corn is a common practice in northern China and is often recommended for small ruminants [32]. Steam flaking served as the intensive benchmark, with exposure to 90°C–100°C steam plus roller pressure achieving over 45% starch gelatinization, a gold standard for beef cattle in many feedlots [31]. Contrasting these four strategies allowed us to examine the relative contributions of particle size, starch gelatinization, and rumen retention time to lamb growth and digestive efficiency.

### Growth performance

The superior ADG of lambs fed the WS diet (319 g/d) compared with GD-fed lambs (257 g/d) aligns with previous studies that larger particle sizes improve ruminant growth relative to finely GD [33,34]. The 24% increase in ADG with WS over GD and the 7% over IT underscore the importance of corn’s physical form in determining nutrient utilization. The 22% higher ADG of IT lambs relative to GD lambs further supports this notion, suggesting that larger particles promote slow fermentation and promote starch bypass to the small intestine [31,33]. Greater bypass starch likely contributes to more efficient energy use, as post-ruminal digestion yields less heat and methane than microbial fermentation [6,35].

While total tract DM digestibility provides an overall measure of nutrient utilization, it does not differentiate among ruminal, small intestinal, or hindgut digestion. The comparable growth performance of IT and WS, despite a 21 g/d difference, suggests that steaming only marginally enhanced starch availability while preserving the benefits of whole grain feeding. Future work quantifying duodenal starch flow would clarify the contributions of ruminal *versus* post-ruminal digestion to these outcomes.

The intermediate growth performance of SF-fed lambs compared to WS- and GD-fed lambs was unexpected, as steam flaking generally increases starch availability and growth in cattle [36,37]. However, the thinner 2.5 mm flake thickness used here may have promoted excessive ruminal starch degradation, leading to energy loss as heat and volatile fatty acids [5]. Lambs’ smaller rumen capacity and faster passage rate could exacerbate this effect, reducing efficiency compared with cattle [38]. Overly rapid fermentation might also increase the risk of subclinical acidosis. These findings emphasize that optimal grain processing parameters are species-specific.

All treatments were pelleted under identical conditions (conditioning at 85°C for 45 seconds, pelleting at 90°C, air cooling, and a ring die compression ratio of 1:7). Thus, performance differences primarily reflect pre-pelleting processing effects rather than pelleting-induced structural changes. While pelleting likely caused partial starch gelatinization, the effect was limited due to short conditioning time and low moisture [39].

Although rumen pH at slaughter did not differ significantly (Table 5), dynamic parameters such as ruminal VFA or lactate concentrations were not monitored. Therefore, potential subclinical acidosis cannot be ruled out. The numerically lower DMI in SF-fed lambs could indicate mild digestive discomfort. Future studies incorporating real-time rumen fermentation profiles would provide a more complete understanding of these physiological responses.

### Nutrient digestibility and metabolic implications

DM digestibility followed expected patterns, with intensive processing generally increasing total-tract digestibility [7]. However, the superior growth observed in WS-fed lambs despite intermediate digestibility suggests that overall total tract DM digestibility does not fully reflect energetic efficiency. The higher ADG with WS likely reflects enhanced small intestinal starch digestion, which is more energy-efficient than ruminal fermentation [40]. The 2.8 percentage-unit higher DMD in WS than in GD (Table 2) supports the idea that WS achieved a favorable balance between ruminal fermentation and post-ruminal utilization.

Because pelleting conditions were uniform, observed differences across treatments likely stemmed mainly from initial corn processing. Future studies could include particle size analyses pre- vs. post-pelleting to confirm this. The high digestibility observed in SF-fed lambs without a corresponding improvement in growth supports the hypothesis that excessive ruminal starch degradation may reduce net energy efficiency by increasing metabolic heat and methane losses [8].

Serum biochemical parameters offered valuable insight into the nutritional and metabolic status of the lambs. The elevated serum TP in GD-fed lambs likely reflects changes in protein metabolism or nutrient supply, as serum TP is generally considered a marker of protein status in ruminants [41]. However, this increase did not correspond to improved growth performance, suggesting that factors such as dietary protein quality, utilization efficiency, or feed intake may have limited growth outcomes. While microbial protein is essential for ruminant nutrition, its contribution to growth depends on amino acid composition and intestinal digestibility [42,43]. Balancing rumen-degradable and rumen-undegradable protein fractions remains critical for optimizing growth performance.

The lower cholesterol concentrations observed in SF-fed lambs may reflect altered lipid metabolism, potentially linked to increased propionate production [44]. Propionate, a key gluconeogenic precursor, regulates hepatic lipid metabolism and cholesterol synthesis [11]. In addition, the significantly lower LDL-C in WS-fed lambs suggests improved lipid utilization and metabolic health, potentially supporting long-term productivity and feed efficiency.

### Rumen microbial ecology

Rumen microbial analysis showed clear shifts at both phylum and genus levels in response to corn processing. The higher Firmicutes:Bacteroidota ratio observed in GD, WS and SF treatments compared with IT is consistent with earlier reports that starch availability modulates this ratio [45]. Firmicutes are well known for their strong starch-degrading capacity [46], and their increased abundance likely reflects greater starch availability in processed diets. The numerically lower Firmicutes:Bacteroidota ratio in WS than in GD and SF may suggest that WS’s intact form of corn likely moderated degradation rates, favoring bypass over excessive ruminal fermentation. These findings align with previous evidence that diets rich in fermentable starch promote Firmicutes proliferation [47].

A notable finding was the sixfold increase in *Erysipelotrichaceae*_UCG-002 in WS-fed lambs. Although the functional role of this taxon is not fully understood, it has been associated with efficient starch metabolism and improved feed efficiency [47,48]. Its enrichment in the rumen of WS-fed lambs may suggest that steam cooking fosters microbial populations capable of optimizing starch utilization, highlighting feed processing as a potential “microbiome engineering” tool.

Minor phyla, including *Acidobacteriota*, *Chloroflexi*, *WPS-2*, and *RCP2-54*, were detected only in IT-fed lambs. These taxa, often associated with plant fiber degradation, may reflect slower fermentation and increased fibrolytic activity, contributing to greater microbial diversity and rumen stability [49].

The microbial profile of SF-fed lambs, characterized by greater abundances of *Pseudoscardovia*, *Megasphaera*, and *Oribacterium*, suggests intensified fermentation. While *Megasphaera* can mitigate acidosis by utilizing lactate, its increased abundance indicates rapid starch fermentation and potentially less efficient glucose absorption [50]. Collectively, these results demonstrate that different processing methods promote distinct microbial “strategies” that ultimately influence host performance.

### Carcass characteristics and meat quality

The trend toward heavier HCW in WS-fed lambs mirrors their superior growth performance, suggesting that the benefits of steam cooking extend through to slaughter yield. In contrast, carcass quality traits such as pH, tenderness, and water-holding capacity were unaffected by processing method, consistent with reports that feed processing affects growth performance rather than meat quality [26]. This finding is practically important, as it indicates that enhanced growth can be achieved without compromising carcass characteristics.

Differences in organ weights also warrant discussion. Larger hearts and kidneys in IT and WS lambs may reflect higher metabolic demand, particularly in IT where increased rumen fermentation could impose greater cardiovascular and renal workload [51,52]. Such adaptations are typical of animals with higher energy turnover or altered rumen fermentation profiles.

### Practical implications and recommendations

Whole steamed corn offers clear advantages for lamb fattening systems. Steam cooking likely gelatinizes starch sufficiently to enhance small intestinal digestibility, while kernel integrity promotes rumen bypass and avoids excessive ruminal fermentation. This combination appears to optimize both energy efficiency and metabolic health. From a production standpoint, steam cooking may also be more economical than steam flaking, as it avoids the additional energy and equipment costs of rolling while still delivering performance benefits. Even the modest numerical gain in ADG with WS over IT (21 g/d) could yield meaningful economic returns across a full feeding cycle. Overall, steam cooking IT kernels before pelleting may represent a practical and cost-effective strategy for improving growth performance in intensive lamb fattening systems.

## CONCLUSION

Whole steamed corn consistently outperformed the other three treatments. It increased ADG by 22% (58 g/d), improved dry matter digestibility by 2.8 percentage units and tended to raise hot-carcass weight by 18% compared with GD. Relative to IT and SF, WS maintained a 7%–24% ADG advantage, equivalent to approximately a 5%–11% improvement in feed efficiency. These performance benefits align with shifts in the rumen microbiome, most notably a higher relative abundance of starch-utilizing Firmicutes and *Megasphaera*, suggesting that the combination of moist heat and large particle size slows ruminal starch release while boosting post-ruminal utilization. Future work should refine steaming conditions (time–temperature combinations), measure site-specific starch flow to confirm bypass contributions, and track long-term health and productivity responses. For lamb finishing diets delivered as pelleted total mixed rations, steam-cooking whole corn kernels before pelleting shows promise as a precision-feeding strategy, but further trials under commercial conditions are warranted.

## Figures and Tables

**Table 1:** Composition and nutrient content of the experimental diet

Item	
Ingredient (kg/t as-fed basis)	
Corn	500
Premix (trace minerals and vitamins)^1)^	20
Corn germ meal	150
Sunflower meal	120
Rice husk	21
Sunflower seed hulls	71
Cottonseed meal	70
Bentonite	20
Limestone	15
Dicalcium phosphate	8
Sodium chloride	5
Analyzed composition (g/kg dry matter [DM])	
DM (g/kg as-fed)	885.5
Organic matter (OM)	915.4
Crude protein (CP)	141.2
Neutral detergent fiber (NDF)	193.2
Acid detergent fiber (ADF)	101.7
Calcium (Ca)	9.7
Phosphorus (P)	5.4
Metabolizable energy (MJ/kg DM)^2)^	9.05

1) Each kilogram of premix contains: 200,000 IU vitamin A, 60,000 IU vitamin D_3_, 550 mg vitamin E, 800 mg niacin, 650 mg Cu (as CuSO_4_), 2,800 mg Fe (as FeSO_4_), 900 mg Mn (as MnSO_4_), 16 mg Se (as Na_2_SeO_3_), 3,600 mg Zn (as ZnSO_4_), 20 mg Co (as CoCl_2_), 15 mg Ca (as CaCO_3_), and 15 g lysine. The carrier consisted of glucose, rice bran, zeolite powder, and limestone powder.

2) Metabolizable energy was estimated from NRC [19].

**Table 2:** Effects of different corn processing methods on lamb growth performance (n = 3 pens per treatment), dry matter intake (n = 3 pens per treatment), and apparent dry matter digestibility (n = 6 lambs per treatment)

Item	Treatment				SEM	p-value

	WS	GD	IT	SF		
Initial body weight (kg)	34.0	34.7	34.8	34.3	0.54	0.772
Final body weight (kg)	53.3	50.2	52.6	49.9	1.21	0.183
Average daily gain (g/d)	319^b^	261^a^	298^ab^	257^a^	14.2	0.013
Relative Average daily gain^1)^	122	100	114	98	-	-
Dry matter intake (kg/d)	1.55	1.49	1.52	1.40	0.061	0.307
Feed-to-gain ratio	4.85^a^	5.71^b^	5.10^ab^	5.43^ab^	0.332	0.044
Dry matter digestibility (%)	72.1^c^	69.3^b^	66.3^a^	74.1^d^	0.63	0.001

1) Relative to GD (set as 100%).

a–d Means within a row with different superscripts differ significantly (p<0.05).

WS, whole steamed corn; GD, ground corn; IT, intact corn; SF, steam-flaked corn; SEM, standard error of the mean.

**Table 3:** Effects of corn processing methods on the blood biochemical parameters of lambs (n = 12 lambs per treatment)

Item	Treatment				SEM	Sampling time		SEM	p-value		
		
	WS	GD	IT	SF		d 31	d 60		T	S	T×S
Protein metabolism											
Total protein (g/L)	69.8^ab^	72.6^b^	67.8^a^	66.8^a^	1.32	67.9	70.7	0.94	0.022	0.074	0.889
Albumin (A) (g/L)	27.0	26.9	26.5	25.5	0.44	25.6	27.4	0.31	0.079	<0.001	0.114
Globulin (G) (g/L)	42.8^a^	45.6^b^	41.1^a^	41.3^a^	1.35	42.1	43.3	0.96	0.093	0.538	0.790
A/G	0.64	0.60	0.66	0.64	0.024	0.62	0.65	0.017	0.267	0.288	0.488
BUN (mmol/L)	9.63	10.08	9.58	10.32	0.450	7.39	12.41	0.318	0.284	<0.001	0.209
Creatinine (μmol/L)	58.9	56.5	52.9	51.4	2.7	-	-	-	0.487	-	-
Energy substrates and enzymes											
Glucose (mmol/L)	4.85	4.97	4.86	4.92	0.144	4.72	5.08	0.104	0.905	0.022	0.360
Triglycerides (mmol/L)	0.119	0.133	0.136	0.084	0.0160	0.121	0.116	0.0114	0.319	0.783	0.059
Cholesterol (mmol/L)	1.86^b^	1.71^b^	1.82^b^	1.47^a^	0.075	1.56	1.87	0.053	0.004	<0.001	0.791
HDL (mmol/L)	0.151	0.114	0.218	0.166	0.0395	0.145	0.179	0.0268	0.288	0.428	0.917
LDL (mmol/L)	0.712^b^	0.614^ab^	0.641^ab^	0.536^a^	0.0383	0.573	0.679	0.0271	0.029	0.010	0.365
α-Amylase (U/L)	18.2	16.9	16.9	11.4	2.14	17.1	14.5	1.55	0.189	0.149	0.083
Lipase (U/L)	11.8	12.6	15.0	9.8	1.36	-	-	-	0.358	-	-
NEFA (mmol/L)	0.087	0.076	0.062	0.121	0.0196	-	-	-	0.290	-	-
Liver function											
ALT (U/L)	17.1	17.7	17.5	16.1	1.02	17.0	17.2	0.75	0.783	0.794	0.949
AST (U/L)	121	124	113	155	17.2	89	167	12.3	0.365	<0.001	0.254
ALP (U/L)	577	504	580	524	51.3	542	550	36.7	0.741	0.859	0.768

a,b Means within a row with different superscripts differ significantly (p<0.05).

WS, whole steamed corn; GD, ground corn; IT, intact corn; SF, steam-flaked corn; SEM, standard error of the mean; T, treatment; S, sampling time; T×S, interaction between treatment and sampling time; BUN, blood urea nitrogen; HDL, high-density lipoprotein cholesterol; LDL, low-density lipoprotein cholesterol; NEFA, non-esterified fatty acids; ALT, alanine aminotransferase; AST, aspartate aminotransferase; ALP, alkaline phosphatase.

**Table 4:** Effects of corn processing methods on the relative abundance of rumen bacteria in the levels of phylum and genus^1)^ (n = 15 lambs per treatment)

Item	Treatment				SEM	p-value

	WS	GD	IT	SF		
Phylum						
Bacteroidota	47.2	45.7	53.4	44.3	4.02	0.427
Firmicutes	39.9^b^	41.3^b^	26.3^a^	42.9^b^	4.12	0.028
Proteobacteria	2.0	5.0	5.3	0.8	2.02	0.369
unidentified_Bacteria	1.7^ab^	0.6^a^	3.9^b^	2.0^ab^	0.79	0.039
Actinobacteriota	2.7^b^	1.2^a^	2.2^ab^	4.6^c^	0.50	0.017
Euryarchaeota	0.5	1.0	1.7	0.5	0.57	0.399
Acidobacteriota	0.0^a^	0.0^a^	1.2^b^	0.0^a^	0.12	<0.001
Spirochaetota	0.2	0.3	0.3	0.2	0.07	0.650
Chloroflexi	0.0^a^	0.0^a^	0.9^b^	0.0^a^	0.10	<0.001
Desulfobacterota	0.2	0.2	0.1	0.2	0.03	0.105
Cyanobacteria	0.1	0.3	0.2	0.2	0.11	0.733
WPS-2	0.0^a^	0^a^	0.2^b^	0.0^a^	0.03	<0.001
RCP2–54	0^a^	0^a^	0.2^b^	0^a^	0.02	<0.001
Genus						
*Erysipelotrichaceae*_UCG-002	14.6^b^	11.2^ab^	2.0^a^	10.5^ab^	3.35	0.066
*Succinivibrionaceae*_UCG-001	1.6^a^	0.6^a^	3.8^b^	0.4^a^	0.69	0.005
*Olsenella*	2.2^b^	0.7^a^	0.9^ab^	1.7^ab^	0.46	0.074
*Pseudoscardovia*	0.2^a^	0.0^a^	0.2^a^	1.9^b^	0.42	0.016
*Rikenellaceae*_RC9_gut_group	1.7^a^	1.4^a^	2.8^b^	1.3^a^	0.33	0.010
*Megasphaera*	0.1^a^	0.0^a^	0.0^a^	0.8^b^	0.15	0.003
*Acidothermus*	0^a^	0^a^	0.3^b^	0.0^a^	0.05	<0.001
*Puia*	0^a^	0^a^	0.2^b^	0^a^	0.03	<0.001
*Oribacterium*	0.2^a^	0.2^a^	0.2^a^	0.3^b^	0.03	0.020
*Acidibacter*	0^a^	0^a^	0.2^b^	0^a^	0.02	<0.001
*Candidatus_Solibacter*	0.0^a^	0.0^a^	0.2^b^	0.0^a^	0.02	<0.001
*Bryobacter*	0^a^	0.0^a^	0.2^b^	0^a^	0.02	<0.001

1) The value is presented only when the relative abundance is over 0.2% of the total bacteria in at least one of the four treatments.

a–c Means within a row with different superscripts differ significantly (p<0.05).

WS, whole steamed corn; GD, ground corn; IT, intact corn; SF, steam-flaked corn; SEM, standard error of the mean.

**Table 5:** Effects of corn processing methods on slaughter performance, organ weight and indices, digestive tract pH, and meat quality of finishing lambs (n = 6 lambs per treatment)

Item	Treatment				SEM	p-value

	WS	GD	IT	SF		
Slaughter performance						
Body weight (BW, kg)	58.4	51.9	54.0	53.7	2.28	0.254
Hot carcass weight (HCW, kg)	27.7	23.4	26.3	26.0	1.10	0.078
Dressing percentage (%)	47.4	45.4	48.9	49.0	2.16	0.626
GR value (mm)^1)^	48.6	45.9	44.3	41.3	7.77	0.926
*Longissimus* muscle area (cm^2^)	14.6	12.9	14.7	15.0	0.90	0.360
Backfat thickness (mm)	1.70	1.47	1.40	1.57	0.125	0.365
Head (kg)	3.92	3.62	3.67	3.68	0.178	0.645
Head/BW (%)	6.7	7.05	6.82	6.91	0.327	0.898
Hoof (kg)	1.67	1.37	1.46	1.47	0.072	0.057
Hoof/BW (%)	2.88	2.76	2.72	2.75	0.140	0.859
Organ weight (g)						
Heart	232^c^	196^a^	243^c^	212^b^	6.9	<0.001
Liver	1219	1015	1107	1081	60.9	0.158
Spleen	152	153	147	132	24.8	0.509
Lung	629	520	683	597	44.2	0.106
Kidney	151^b^	136^ab^	167^b^	127^a^	8.5	0.018
Organ index (%)						
Heart	0.397^a^	0.381^a^	0.452^b^	0.398^a^	0.0154	0.025
Liver	2.10	1.98	2.06	2.02	0.090	0.798
Spleen	0.265	0.205	0.270	0.258	0.0498	0.779
Lung	1.082	1.025	1.276	1.13	0.1055	0.398
Kidney	0.259^a^	0.265^a^	0.309^b^	0.238^a^	0.0150	0.024
pH value						
Rumen	6.38	5.88	6.12	5.99	0.197	0.348
Duodenum	5.45	5.57	6.03	4.92	0.279	0.079
Jejunum	6.08	5.78	6.10	5.78	0.154	0.331
Ileum	6.99	6.69	6.64	6.90	0.257	0.756
Cecum	6.65	6.44	6.64	6.30	0.232	0.659
Meat quality						
Score	5.00	5.17	5.33	5.67	0.202	0.153
Marbling score	1.33	2.00	1.67	1.33	0.266	0.266
Tenderness (N)	58.3	52.2	52.1	48.7	4.43	0.508
Cooked meat yield (%)	57.9	57.9	59.9	58.8	1.23	0.634

1) GR value refers to a measurement of subcutaneous fat depth taken over the 12th rib, 11 cm from the midline of the carcass.

a–c Means within a row with different superscripts differ significantly (p<0.05).

WS, whole steamed corn; GD, ground corn; IT, intact corn; SF, steam-flaked corn; SEM, standard error of the mean.

**Table 6:** Effects of different corn processing methods on rumen morphology in lambs (n = 6 lambs per treatment)

Item	Treatment				SEM	p-value

	WS	GD	IT	SF		
Rumen wall thickness (mm)	1.33	1.47	1.23	1.42	0.114	0.495
Papillae number (/cm^−2^)	60.2	57.9	63.7	55.2	5.52	0.740
Papillae length (cm)	4.20	4.84	3.68	4.83	0.521	0.356
Papillae width (mm)	1.83	1.84	1.89	2.04	0.128	0.636
Reticulum wall thickness (mm)	1.93	1.78	1.97	1.84	0.140	0.778

WS, whole steamed corn; GD, ground corn; IT, intact corn; SF, steam-flaked corn; SEM, standard error of the mean.

## Data Availability

Upon reasonable request, the datasets of this study can be made available from the corresponding author.
